# Sulforaphane Potentiates Anticancer Effects of Doxorubicin and Cisplatin and Mitigates Their Toxic Effects

**DOI:** 10.3389/fphar.2020.00567

**Published:** 2020-05-01

**Authors:** Cinzia Calcabrini, Francesca Maffei, Eleonora Turrini, Carmela Fimognari

**Affiliations:** Department for Life Quality Studies, Alma Mater Studiorum-Università di Bologna, Rimini, Italy

**Keywords:** sulforaphane, doxorubicin, cisplatin, anticancer effects, Nrf2, chemosensitization, chemoresistance, toxicity

## Abstract

The success of cancer therapy is often compromised by the narrow therapeutic index of many anticancer drugs and the occurrence of drug resistance. The association of anticancer therapies with natural compounds is an emerging strategy to improve the pharmaco-toxicological profile of cancer chemotherapy. Sulforaphane, a phytochemical found in cruciferous vegetables, targets multiple pathways involved in cancer development, as recorded in different cancers such as breast, brain, blood, colon, lung, prostate, and so forth. As examples to make the potentialities of the association chemotherapy raise, here we highlight and critically analyze the information available for two associations, each composed by a paradigmatic anticancer drug (cisplatin or doxorubicin) and sulforaphane.

## Introduction

A promising strategy to improve the efficacy of anticancer therapy is the association of chemotherapeutic drugs with natural compounds (Farzaei et al., 2016; Negrette-Guzman, 2019). Indeed, in tumor tissues, phytochemicasl may interact with multiple molecular targets and potentiate the efficacy of traditional anticancer drugs. Moreover, they might exert a protective role against side effects caused by chemotherapeutic agents on off-target tissues.

Sulforaphane (SFN) is a natural isothiocyanate extensively studied for its pleiotropic activity on different cancer models. SFN has been found to exhibit cytotoxic and cytostatic activities through several mechanisms. The production of reactive oxygen species (ROS) is one of the most important. SFN-induced ROS generation promotes the activation of both intrinsic and extrinsic apoptotic pathways. SFN can also cause cell-cycle arrest in tumor cells, partly dependent on the modulation of epigenetic mechanisms including histone acetylation and DNA methylation (Brioness-Herrera et al., 2018). Its activity has been reported even in the most advanced stages of cancer development, where it inhibits pathways involved in metastasis and angiogenesis (Sestili and Fimognari, 2015; Negrette-Guzman, 2019). A very recent study reported that the anticancer activity of SFN involves microRNAs (miRNAs) regulation. miRNAs are post-transcriptional regulators of genes implicated in critical cellular pathways, including apoptosis, cell cycle, and cell differentiation (Rafiei et al., 2020).

A peculiar characteristic of SFN is its ability to exert dichotomous effects. Indeed, SFN is also an indirect ROS scavenger: it up-regulates phase II biotransformation enzymes by enhancing Nuclear factor E2-related factor 2 (Nrf2) activity. SFN disrupts the link between Nrf2 and its suppressor Kelch-like ECH-associated protein 1 (Keap1) and promotes the cytoplasmic and nuclear accumulation of Nrf2 (Briones-Herrera et al., 2018). In the nucleus, Nrf2 acts as a transcription activator for DNA sequences known as antioxidant response elements (ARE). SFN *via* Nrf2 increases the expression of some ARE-target genes including NADPH-quinone oxidoreductase 1 (NQO1), heme-oxygenase (HO-1), and glutamate-cysteine ligase catalytic subunit (GCLC).

In this mini-review, we highlight and critically analyze the available evidence on the anticancer and cytoprotective effects of SFN in association with two paradigmatic anticancer drugs, i.e., doxorubicin (Doxo) and cisplatin (CIS).

## SFN and Doxo Association

### SFN Enhances the Anticancer Efficacy of Doxo

The anthracycline Doxo induces DNA damage through different mechanisms such as topoisomerase II inhibition, generation of ROS, and DNA adduct formation. Doxo undergoes bioreductive activation by redox-cycling reactions, forming a reactive semiquinone. The semiquinone radical intercalates in DNA duplex and generates ROS. ROS increase DNA damage resulting in cytotoxic and cytostatic events (Agudelo et al., 2014). Of note, the generation of ROS is a double-edged sword. It is the key mechanism through which Doxo induces tumor cell death but, at the same time, it may contribute to Doxo toxicity (Angsutararux et al., 2015; Karasawa and Steyger, 2015) and prompt signals leading cancer cells to escape apoptosis (Alimbetov et al., 2018).

**Figure 1:**
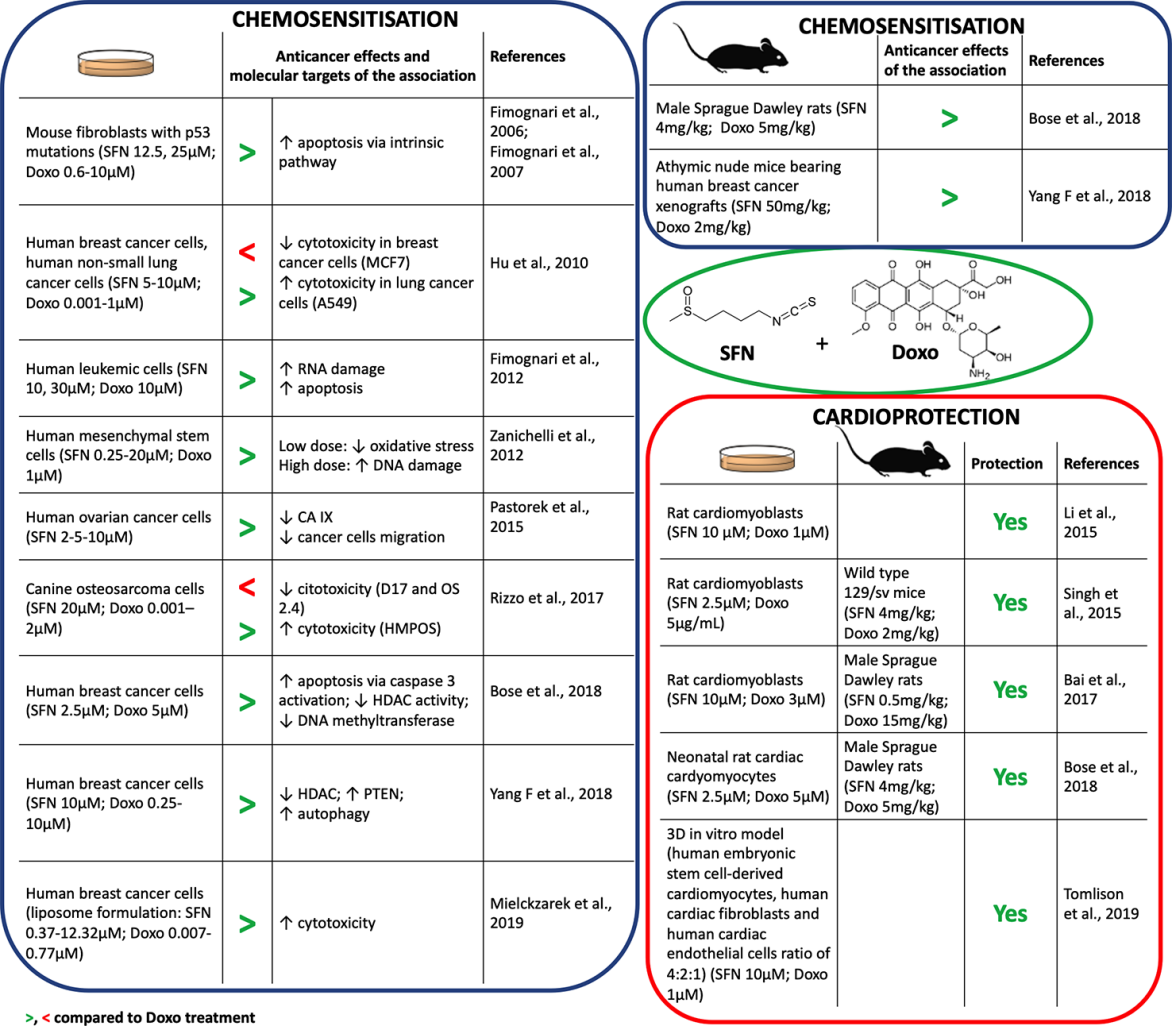
Chemosensitization and cardioprotection of sulforaphane (SFN) in association with doxorubicin (Doxo).

In combination with Doxo, SFN increased its proapoptotic activity in different cell lines (Fimognari et al., 2012; Bose et al., 2018; Mielczarek et al., 2019) (**Figure 1**). Furthermore, SFN reverted Doxo-resistant phenotype in p53-mutated cells, inducing apoptosis irrespective of p53 status (Fimognari et al., 2006; Fimognari et al., 2007). SFN potentiated also the RNA-damaging activity of Doxo, increasing its proapoptotic potential (Fimognari et al., 2012). Besides, SFN improved the sensitivity to Doxo by inducing autophagy *via* epigenetic mechanisms. In particular, SFN suppressed histone deacetylase HDAC6 that in turn activates PTEN (phosphatase and tensin homolog), a tumor suppressor gene and key regulator of autophagy (Yang F, et al., 2018). However, in certain cancer cell lines, SFN showed a hormetic biphasic response. At low doses, it reduced Doxo-induced oxidative stress, but at higher doses it exhibited synergistic effects and promoted DNA damage (Zanichelli et al., 2012) (**Figure 1**).

Some anticancer drugs can lose their efficacy in a hypoxic cancer microenvironment (Muz et al., 2015). The master genes orchestrating molecular response to hypoxia are HIF1α (hypoxia-inducible factor 1α) and its downstream targets, such as carbonic anhydrase protein IX (CA IX). CA IX protein protects from pH imbalance provoked by hypoxia and facilitates invasion and migration of tumor cells (Tafreshi et al., 2014). SFN down-regulated the expression of HIF1α and CA IX proteins in ovarian cancer cells cultivated in hypoxia and reduced their migration (Pastorek et al., 2015) (**Figure 1**). Since HIF1α was found to be upregulated in tumor cells after Doxo treatment (Cao et al., 2013), the down-regulation of HIF1α by SFN could represent a relevant mechanism to enhance Doxo efficacy in cancer cells.

However, conflicting data on the effects of SFN when used in association with Doxo impose caution. Rizzo and coworkers showed that SFN can decrease Doxo's antitumor potential depending on the specific redox status of the cell line (Rizzo et al., 2017). SFN sensitized cells characterized by high basal Nrf2 expression to Doxo, whereas it reduced Doxo's anticancer effects in cells with very low Nrf2 basal levels (Hu et al., 2010) (**Figure 1**). Thus, the effects of SFN+Doxo may depend on the Nrf2 basal level of tumor cell type. Of note, most of the data on SFN+Doxo effects was obtained by *in vitro* studies. Evidence has started to accrue *in vivo* (**Figure 1**) and confirmed the synergistic effect of the association. The association of SFN could thus allow the use of lower doses of Doxo and a reduction of its adverse effects. Accordingly, Bose and coworkers demonstrated that the effective dosage of Doxo could be lowered by 50% in combination with SFN (Bose et al., 2018). Altogether, data on SFN-Doxo association are promising, but not conclusive.

### SFN Mitigates Doxo-Induced Cardiotoxicity

The most common adverse effect in patients receiving Doxo-based chemotherapy is cardiotoxicity. The mechanism of Doxo cardiotoxicity is multifactorial. It includes ROS-mediated myocardium injury, impaired mitochondrial function, cardiomyocyte apoptosis, and dysregulation of Ca^2+^ homeostasis. All together these events lead to an increased rate of heart failure (Bai et al., 2017; Tomlinson et al., 2019).

Several *in vitro* studies showed the cardioprotective effects of SFN after pre- or co-treatment with Doxo (**Figure 1**). SFN contrasted Doxo-induced oxidative stress and cardiomyocytes' death. In particular, SFN prevented apoptosis inhibiting: i) the activation of Bax protein, ii) the release of cytochrome c, iii) the activation of caspase-3, iv) the loss of mitochondrial transmembrane potential, and v) the generation of mitochondrial ROS (Li et al., 2015; Singh et al., 2015). SFN cardioprotection was mediated by Nrf2 activation and the subsequent induction of phase II enzymes, such as HO-1 (Li et al., 2015). Interestingly, Tomlison and colleagues confirmed the pivotal role of Nrf2 in a 3D model exhibiting key features of cardiac tissue (**Figure 1**). Using this model, inducers of Nrf2, including SFN, exploited cardioprotective activity similar to dexrazoxane, used in patients receiving high cumulative dose of anthracyclines (McGowan et al., 2017). Similarly, SFN counteracted oxidative damage and heart failure induced by Doxo *in vivo* (**Figure 1**). In particular, SFN activated cardiac Nrf2 and upregulated its downstream targets, including genes involved in glutathione (GSH) synthesis, HO-1, and NQO1 (Singh et al., 2015; Bai et al., 2017; Bose et al., 2018). The reduction of Doxo-induced myocardial injury markers, such as creatine kinase-MB, aspartate aminotransferase, lactate dehydrogenase, and troponin I, further support the cardioprotective activity of SFN (Singh et al., 2015; Bai et al., 2017).

Doxo strongly compromised mitochondrial activity, due to its conversion by the mitochondrial complex I of the electron transport chain (ETC) into the more reactive semiquinone (Bose et al., 2018). SFN preserved ETC functionality and mitochondria ultrastructure of cardiac cells from oxidative stress damage in Doxo-treated animal models (Singh et al., 2015; Bose et al., 2018).

Fibrosis and inflammation can contribute to heart stiffness and dysfunction. SFN prevented Doxo-induced cardiac fibrosis inhibiting cardiac collagen accumulation and contrasting the up-regulation of connective tissue growth factors induced by Doxo (Bai et al., 2017). Moreover, it decreased Doxo-induced inflammatory heart markers, such as plasminogen activator inhibitor-1 (Bai et al., 2017) and serum levels of IL-6 and TNF-α (tumor necrosis factor-α) (Bose et al., 2018).

Finally, SFN led to an increased survival rate in animals co-treated with SFN+Doxo compared to those treated with Doxo (85% reduction in rats and 90% reduction in mice in hazard of dying from Doxo exposure) (Singh et al., 2015; Bose et al., 2018). This evidence is mainly imputable to the preservation of heart functionality (measured by ejection fraction, fractional shortening, and stroke volume) mediated by SFN.

On the whole, *in vitro* mechanistic studies and *in vivo* results univocally outline SFN as a promising molecule to prevent Doxo-induced cardiotoxicity.

## SFN and CIS Association

### SFN Enhances the Anticancer Efficacy of CIS

CIS is a platinum derivative used for both solid and liquid cancer treatment (Volarevic et al., 2019). Its anticancer activity is due to multiple mechanisms involving binding to genomic or mitochondrial DNA to generate DNA damage and interfering with DNA repair systems, eventually leading to activation of p53 and induction of apoptosis. CIS-induced DNA damage is also due to its ability to generate ROS (Ghosh, 2019). Thus, compounds able to increase ROS or DNA damage could enhance CIS anticancer effects.

**Figure 2:**
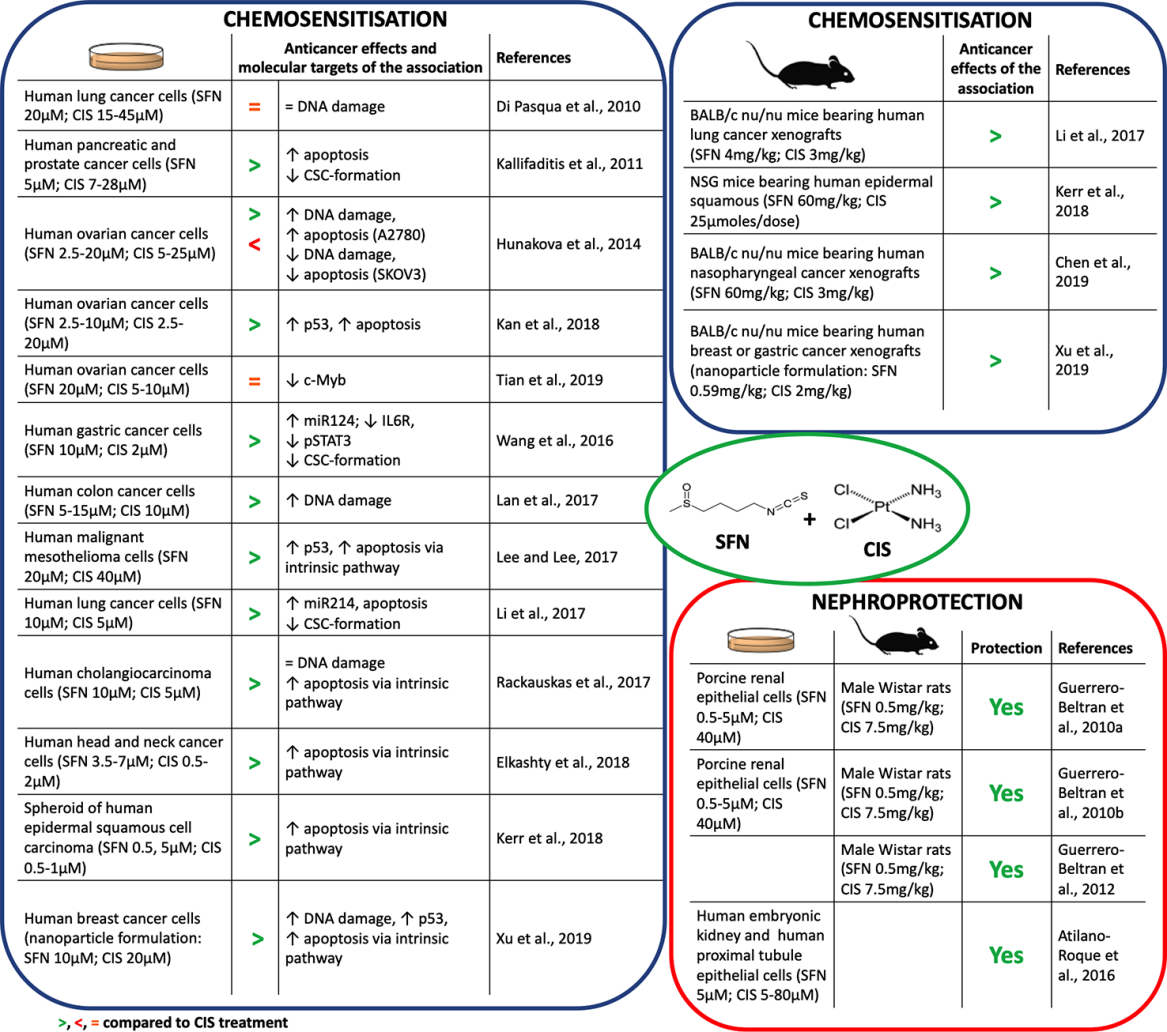
Chemosensitization and nephroprotection of sulforaphane (SFN) in association with cisplatin (CIS).

Many studies reported that SFN synergizes with CIS in counteracting cancer development (**Figure 2**). SFN enhanced CIS-induced DNA damage and apoptosis in many cancer cell lines (Hunakova et al., 2014; Lan et al., 2017; Lee and Lee, 2017; Elkashty et al., 2018; Kan et al., 2018; Xu et al., 2019). In most of them apoptosis occurred *via* p53 and caspases activation. Few studies, however, deepened the mechanisms involved in those effects. The SFN's ability to inhibit DNA repair (Piberger et al., 2014) or to transiently depletes GSH (Pappa et al., 2007) represent two candidate mechanisms. In particular, GSH depletion lowered the inactivation/excretion of CIS that occurs mainly *via* conjugation with GSH or metallothioneins (Ghosh, 2019). Accordingly, a nanoparticle delivery system containing SFN plus CIS decreased GSH levels and enhanced the intracellular levels of CIS (Xu et al., 2019). Since GSH depletion deprives cells of one of the most important defenses against oxidative stress, a possible consequence could be an increase in ROS and an enhanced CIS-induced DNA damage. The role of ROS generation on the cytotoxicity of SFN+CIS was defined pre-treating cancer cells with N-acetylcysteine (NAC), a GSH precursor. NAC prevented ROS generation, activation of the mitochondrial apoptotic pathway as well as cell-cycle arrest and autophagy induced by SFN+CIS (Lee and Lee, 2017). Similarly, NAC abrogated the antitumor activity of SFN+CIS nanoparticles *in vivo* (Xu et al., 2019) (**Figure 2**).

Interestingly, SFN can increase the cytotoxicity of CIS also through mechanisms different from DNA damage. The association reduced the CIS-induced overexpression of antiapoptotic proteins such as Bcl2 (Rackauskas et al., 2017) (**Figure 2**), an event involved in the onset of chemoresistance (Galluzzi et al., 2012).

Another mechanism of CIS chemoresistance is the formation of cancer stem cells (CSC) (Wang et al., 2016). Both in *in vitro* and *in vivo* models, CIS-resistant cells overexpress β-catenin and c-Myc proteins, which are involved in CSC self-renewal (Li et al., 2017). CIS+SFN reduced the CSC population and inhibited their stem-like cell properties and viability in many cancer cells (Kallifaditisis et al., 2011; Wang et al., 2016; Li et al., 2017) (**Figure 2**). SFN reduced the activation of β-catenin/c-Myc pathway through the up-regulation of miR-214, a negative post-translational regulator of both c-Myc and β-catenin (Li et al., 2017). Through the up-regulation of one more miRNA, i.e., miR-124 targeting the IL-6 receptor gene (Xiao et al., 2015), SFN counteracted CIS-activation of IL-6/STAT3 pathway, which seems to be involved in CIS-induced expansion of CSC cells (Wang et al., 2016). STAT3 signaling is also activated by c-Myb, a protein associated with CIS resistance and CSC self-renewal (Zhang et al., 2012). SFN reverted c-Myb-induced cancer cell proliferation and invasion and sensitized cells to CIS (Tian et al., 2019).

In summary, many reports disclose the ability of SFN to enhance CIS's anticancer activity and counteract chemoresistance, although there are some exceptions. As an example, SFN did not enhance CIS's cytotoxicity in a lung cancer cell line. Tubulin-binding drugs are widely used with CIS to enhance its cytotoxicity in non-small cell lung cancer. SFN, if compared with other isothiocyanates, weakly depletes β-tubulin levels (Di Pasqua et al., 2010). This evidence could explain its lack of activity in those cells. Besides, SFN exhibited a controversial role in two ovarian cancer cell lines: it synergized with CIS in A2780 cells and antagonized CIS effects in SKOV3 cells (Hunakova et al., 2014). A2780 cells have a weakly efficient Nrf2 pathway and cannot restore the depletion of GSH induced by SFN. Thus, the association significantly increased DNA damage and apoptosis compared to CIS alone. Conversely, SKOV3 cells have a highly efficient Nrf2 pathway. Thus, SFN-induced activation of the Nrf-2 pathway protected SKOV3 cells from the cytotoxicity of CIS instead of sensitizing them to CIS (Hunakova et al., 2014).

### SFN Mitigates CIS-Induced Nephrotoxicity

CIS therapy causes nephrotoxicity in 30–40% of patients (Volarevic et al., 2019). The mechanism behind the onset of nephrotoxicity is particularly complex and involves multiple mechanisms, including ROS generation, mitochondrial dysfunction, apoptosis, necrosis, and autophagy of renal cells. Moreover, inflammation exacerbates these processes (Holditch et al., 2019).

ROS generation and mitochondrial dysfunction represent the earliest events in CIS-induced nephrotoxicity. SFN reduced CIS-induced ROS generation *in vitro*. It increased GSH pool and antioxidant enzyme activity, and reduced markers of nitrosative and oxidative stress. Accordingly, SFN ameliorated cellular, plasma, kidney, and liver oxidative status (Guerrero-Beltran et al., 2010a; Guerrero-Beltran et al., 2010b; Atilano-Roque et al., 2016) (**Figure 2**). Furthermore, SFN improved renal histopathology and physiological functions in rats treated with CIS (Guerrero-Beltran et al., 2010a; Guerrero-Beltran et al., 2010b). SFN antioxidant activity takes place through the Nrf2 pathway. Treatment with SFN before CIS exposure activated the Nrf2 pathway and its target genes (i.e., GCLC and NQO1) and protected from CIS-induced renal cell injury (Guerrero-Beltran et al., 2010a; Atilano-Roque et al., 2016). The inhibition of GCLC and NQO1 nullified nephroprotection (Guerrero-Beltran et al., 2010a). This finding clearly points out the close link between the SFN-protective effect and its ability to activate the Nrf2 pathway.

CIS accumulates in mitochondria (Dzamitika et al., 2006) and depletes GSH levels, thus increasing mitochondrial oxidative stress and damage to complex I (Guerrero-Beltran et al., 2010a). ROS exacerbate complex I damage and activate several pathways involved in apoptosis or inflammation (Sharma et al., 2009). SFN prevented CIS-induced alterations of mitochondrial functionality in rat kidney (Guerrero-Beltran et al., 2010a) and counteracted the pathways activated by ROS in CIS-induced kidney damage (Guerrero-Beltran et al., 2012). In particular, SFN increased pro-survival ERK (extracellular signal-regulated kinase) and antiapoptotic p38β mitogen-activated protein kinase, and decreased the proapoptotic Jun N-terminal kinase (JNK) and p38α pathways. SFN was also able to decrease TNF-α, nuclear factor kappa-light-chain-enhancer of activated B cells (NF-κB), adhesive molecule expression, and leukocytes and macrophage recruitment into renal tissue and reduce kidney inflammation (Guerrero-Beltran et al., 2012) (**Figure 2**).

These findings highlight the pivotal role of oxidative stress in CIS toxicity and the ability of SFN of counteracting these events through its antioxidant properties.

## Conclusions

Pre-clinical existing data highlight that SFN enhances the anticancer activity of Doxo and CIS and counteracts the off-target toxicity through multiple mechanisms. In particular, SFN strongly activates the Nrf2 antioxidant signaling pathway. This evidence could have clinically relevant implications for cancer therapy as Nrf2 activation in cancer cells may contribute to the onset of either chemosensitisation or chemoresistance (Bai et al., 2016; Catanzaro et al., 2017). Most anticancer drugs amplify ROS levels in cancer cells over a threshold to induce cell death and tumor regression. Anthracyclines produce the highest levels of cellular ROS; alkylating drugs, platinum-based drugs, camptothecins, arsenic-based drugs, and topoisomerase inhibitors generate high levels of ROS; taxanes, Vinca alkaloids, nucleotide analogues, and antimetabolites induce lower levels of ROS (Yang H, et al., 2018). This means that the effect of SFN when used in association with anticancer therapy could be not easily predicted, and indeed, even with the two anticancer drugs included in our review, the effects of SFN are sometimes discordant, as reported above.

In addition, it is well known that cancer cells are not homogeneous. Reprogramming of cancer cells impacts on disease's progression and contributes to their heterogeneity (Milkovic et al., 2017). As an example, in later stages of cancer, Nrf2 and Keap1 are mutated and Nrf2 activity increased. This means that inhibitors of Nrf2 could be better than activators of Nrf2 in the later stages of the disease. Thus, cancer stage should be taken into account for the usage of specific Nrf2 activators or inhibitors during cancer therapy.

Of note, Nrf2 modulation was observed in women orally treated with a broccoli sprout preparation containing 200 µmol of SFN/g. In their breast tissues, increased NQO1 and HO-1 transcripts and NQO1 enzymatic activity have been found (Cornblatt et al., 2007). A phase-II clinical trial is actually recruiting patients with the aims to investigate the ability of SFN-rich broccoli sprout extracts to (i) enhance Doxo anticancer effects on women with breast cancer undergoing neoadjuvant chemotherapy, with no prior cardiac disease and who will receive Doxo without Her-2 receptor antagonists as part of their clinical care, (ii) protect from Doxo-associated cardiac dysfunction, and (iii) explore the role of Nrf2 in this association therapy (ClinicalTrial.gov identifier: NCT03934905, 2019). This interventional study will certainly contribute to define the role of Nrf2 modulation in the efficacy and safety of SFN when associated with traditional anticancer therapies.

Concerning the safety profile of SFN in association with anticancer drugs, SFN prevented changes in animal body weight caused by either Doxo or CIS (Singh et al., 2015; Kerr et al., 2018; Chen et al., 2019). However, a recent study recorded hematic signs of possible myelosuppression and hepatotoxicity in animals exposed to CIS+SFN. Interestingly, these side effects became negligible when drugs were delivered in nanoparticles (Xu et al., 2019), a formulation improving the release of drugs in tumor cells. Thus, a controlled delivery system may enhance chemotherapy efficacy and reduce systemic toxicity of SFN+CIS.

Last but not least, the stability and bioavailability of SFN may influence its chemosensitizing/chemoprotective effects. Thus, the pharmacokinetics of SFN in association with anticancer drugs should be addressed to fully understand its clinical potential in the oncological field.

## Author Contributions

CC, FM, ET, and CF analyzed the scientific literature. CC, FM, and ET wrote the manuscript. CF designed the study and revised the manuscript.

## Conflict of Interest

The authors declare that the research was conducted in the absence of any commercial or financial relationships that could be construed as a potential conflict of interest.
